# Comprehensive assessment and progression of health status during neurorehabilitation in survivors of critical illness: a prospective cohort study

**DOI:** 10.1186/s13613-024-01396-x

**Published:** 2024-11-26

**Authors:** Marion Egger, Melanie Finsterhölzl, Daria Farabegoli, Franziska Wippenbeck, Maria Schlutt, Friedemann Müller, Volker Huge, Klaus Jahn, Jeannine Bergmann

**Affiliations:** 1 Department of Neurology, Schoen Clinic Bad Aibling, Research Group, Kolbermoorer Strasse 72, 83043 Bad Aibling, Germany; 2 Institute for Medical Information Processing, Biometry, and Epidemiology (IBE), Faculty of Medicine, LMU Munich, Pettenkofer School of Public Health, Munich, Germany; 3 Department of Critical Care Medicine and Anesthesiology, Schoen Clinic Bad Aibling, Bad Aibling, Germany; 4 Department of Anesthesiology, LMU University Hospital, LMU Munich, Munich, Germany; 5 German Center for Vertigo and Balance Disorders, LMU, University Hospital, Munich, Germany

**Keywords:** Critical care, Critical illness, Intensive Care Units, ICU-acquired weakness, Neurological rehabilitation, Outcome, Patient-reported outcomes, Post-intensive care syndrome

## Abstract

**Background:**

Critical illness survivors frequently suffer from long-term impairments, often described as post-intensive care syndrome (PICS). PICS encompasses physical, cognitive, and mental impairments. Additionally, the term intensive care unit (ICU)-acquired weakness (ICUAW) was coined for muscle weakness after critical illness. Research on the progression and outcome of individuals affected by PICS and ICUAW is scant. Thus we aimed to assess the health status and its progression during neurorehabilitation in critically ill patients using comprehensive outcome measures, describe the prevalence of PICS, and evaluate factors associated with rehabilitation outcomes.

**Methods:**

Patients with mixed reasons for critical illness who received ≥ 5 days of mechanical ventilation on the ICU and who were admitted to neurorehabilitation, were eligible to be included in this prospective cohort study. A number of outcomes (patient-reported, clinician-reported, and performance) were assessed after discharge from the ICU (V1) and shortly before discharge from inpatient neurorehabilitation (V2). The prevalence of PICS, defined as having at least one impairment in any PICS dimension), was calculated at V1 and V2. Multiple logistic regressions were conducted to identify factors associated with rehabilitation outcome (poor outcome = modified Rankin Scale > 2) and ICUAW at V2 (MRC sum score < 48).

**Results:**

In total, 250 critical illness survivors (62 ± 14 years, 34% female, median stay on ICU 55 days, median inpatient rehabilitation 65 days) were included. 11 participants (4.4%) died before V2. All outcomes improved significantly during rehabilitation except sensory impairment and pain. PICS was present in 96% at V1 and in 85% at V2, whereby mainly the physical domain (V1: 87%, V2: 66%; ICUAW with MRC sum score < 48) and the cognitive domain (V1:65%, V2:55%; Montreal Cognitive Assessment < 26) were affected. Mental impairment was lower (V1:48%, V2:29%; Hospital Anxiety and Depression Scale > 7), but still affected a considerable number of participants. Accordingly, health-related quality of life was rather low at discharge (0.64 ± 0.28, index value of EQ-5D-5L). MRC sum score at V1, duration of mechanical ventilation, and female gender were significantly associated with a poor rehabilitation outcome. Grip strength in % of reference at V1, age, female gender, and comorbidities were significantly associated with persistent ICUAW at discharge.

**Conclusions:**

Despite significant improvements during rehabilitation, survivors after critical illness experience a substantial burden of PICS and ICUAW at discharge from rehabilitation care. Survivors of critical illness require long-term follow-up, supportive structures, and tailored long-term multi-disciplinary therapies even after intensive rehabilitation.

*Trial registration*: German Clinical Trials Register, DRKS00021753. Registered 03 September, 2020. https://drks.de/search/en/trial/DRKS00021753.

**Supplementary Information:**

The online version contains supplementary material available at 10.1186/s13613-024-01396-x.

## Background

Progress in critical care medicine has substantially increased the probability of surviving life-threatening illnesses [1]. However, the short- and long-term outcomes of intensive care unit (ICU) survivors are often characterized by impairments, which restrict their independence, diminish their health-related quality of life, and hinder their return to their living and working situations [2, 3]. A frequent consequence of critical illness is the post-intensive care syndrome (PICS) [4, 5]. PICS affects up to 80% of ICU survivors and describes the combination of physical, mental, and cognitive impairments [2, 6]. Known risk factors for PICS are, among others, older age, female sex, delirium, and high disease severity [5]. PICS decreases long-term survival and has been reported to persist 1 year after discharge from the ICU in 50% of affected persons, often enduring for even longer periods [6–9]. ICU-acquired weakness (ICUAW) is the major complication arising in the physical domain of PICS, and in turn affects up to 80% of ICU survivors [10]. The term ICUAW was coined to describe a profound muscle weakness in critically ill patients, which is primarily caused by critical illness polyneuropathy (CIP), -myopathy (CIM) or their co-occurrence [11]. Prolonged duration of mechanical ventilation, sepsis, and multiple organ system failure are risk factors for the development of an ICUAW [12, 13]. ICUAW is associated with increased mortality [14], persisting disability, and limited health-related quality of life [15, 16].

Although negative consequences after ICU treatment are commonly observed, many areas of uncertainty exist, as previous studies often neglected important aspects [17]. These include the impact of the patient’s pre-admission status, the need for long-term follow-up, and a lack of well-defined, validated outcome measures [18]. Furthermore, effective multi-disciplinary rehabilitation strategies are urgently needed, but knowledge of treatment beyond the ICU is currently limited [19–22]. Consequently, the effect of post-ICU therapies on long-term outcomes are uncertain [17, 18, 23, 24].

Knowledge of factors associated with ICU outcomes is crucial for developing optimal strategies for prevention and treatment [5]. However, risk factors related to the occurrence of ICUAW, have been evaluated only during the ICU stay [25]. Similarly, factors associated with poor rehabilitation outcomes are currently missing. Identifying variables associated with persistent ICUAW and poor rehabilitation outcomes could aid in tailoring rehabilitation approaches, improving prognosis, and planning post-hospital care.

The prospective cohort study CINAMOPS (Critical Illness Polyneuropathy and Myopathy: Outcomes, Predictors and Longitudinal Trajectories) was designed to address the research gaps described and enhance the understanding of the long-term sequelae after ICU treatments, such as PICS and ICUAW [26]. Within this project, survivors of critical illness are evaluated comprehensively during neurological rehabilitation and up to 2 years after disease onset [26].

Using a large set of patient- and clinician-reported outcomes as well as performance outcomes, the primary aim of this analysis was to comprehensively assess the health status and its progression during neurorehabilitation of critical illness survivors. Furthermore, we aimed to assess the PICS prevalence, and to explore factors associated with rehabilitation outcome and persistent ICUAW. Improved knowledge on the clinical course of ICU survivors is the prerequisite for the development and evaluation of interventions in the future.

## Methods

### Study population and setting

This analysis is part of the single-center, prospective cohort study CINAMOPS, which is currently being conducted at the Schoen Clinic Bad Aibling, Germany. The hospital is a centre for inpatient neurorehabilitation with a focus on critically affected patients (ICU, early neurorehabilitation). Details of the study were previously described [26].

Adult patients (≥ 18 years) who were mechanically ventilated in the ICU for at least 5 days were eligible for the CINAMOPS study. Patients were recruited on admission to neurorehabilitation, after discharge from ICU. Exclusion criteria were (1) palliative care, (2) neuromuscular or neurologic diseases/syndromes causing muscular weakness (e.g. Guillain-Barré syndrome, mysasthenia gravis, porphyria, Lambert-Eaton syndrome, amyotrophic lateral sclerosis, severe autoimmune neuropathy, cervical myelopathy, botulism; in accordance with [27]), (3) insufficient communication abilities (German language skills or cognition) interfering with answering the questionnaires, (4) no muscular weakness (i.e. muscle strength according to the Medical Research Council (MRC) scale 5/5).

During the study, patients received inpatient neurological rehabilitation of individual length with approximately 100 minutes of multi-disciplinary functional therapies per day, including physiotherapy, occupational, dysphagia, and breathing therapies, as well as neuropsychology.

The study was approved by the medical ethics committee of the Ludwig-Maximilians-Universität in Munich according to the Declaration of Helsinki (project no. 20-166). Written informed consent was obtained from all participants (or their legal guardians). The project CINAMOPS was prospectively registered at the German Clinical Trials Register (DRKS00021753). The reporting of this study adhered to the Strengthening the Reporting of Observational Studies in Epidemiology (STROBE) guidelines.

### Study visits and outcomes

The first study visit (V1) took place after admission to neurorehabilitation (a median of 14 days after discharge from ICU), the second study visit (V2) was conducted shortly before discharge from inpatient neurorehabilitation. In order to comprehensively assess the health status, the study visits included a variety of established patient-reported outcomes, performance outcomes, and clinician-reported outcomes, which are displayed in Table 1.

**Table 1:** Overview of clinical outcome assessments

Patient-reported outcomes	
Fatigue Severity Scale-7 (FSS-7)	This assessment is used to evaluate fatigue. The seven-item version was used, as it was demonstrated to have better psychometric properties than the nine–item version [28]. Score: 1–7. The cut-off ≥ 4 was interpreted as indicative of fatigue [29]
Hospital Anxiety and Depression Scale (HADS)	This valid and reliable tool measures anxiety and depression and was repeatedly used in critically ill patients [30]. Score: 0–21 each for anxiety and depression. A score of > 7 in each category was interpreted as clinically significant [31]
EuroQol-5 dimensions-5 level (EQ-5D-5L)	The internationally wide-spread scale is used to measure health-related quality of life [32]. The index value for the German population ranges from -0.205 (0 = health state equivalent to death; negative values = health state worse than death) to 1.000 (best health state) [33]. Patients who died after V1 were assigned a score of 0 in all further study visits. Additionally, the visual analogue scale (included in the EQ-5D-5L; 0–100) was used. 100 indicates the best imaginable state of health
Pain / sensory disturbances	Presence of pain and sensory disturbances: patients were asked to report any experiences of pain and sensory disturbances
Clinician-reported outcomes	
Clinical Frailty Scale	Frailty was assessed by this scale [34], whereby the score ranges from 1 to 9 and 9 indicates deathly ill. A preclinical value was recorded retrospectively at V1
Modified Rankin Scale	This scale describes the overall disability [35]. The score ranges from 0 to 6, whereby 6 indicates death. A preclinical value was recorded retrospectively at V1
Barthel-Index	The Barthel Index [36] is widely used and describes the patients´ independence in activities of daily living like washing, grooming, climbing stairs, toilet use etc. It is a reliable and valid tool for patients after critical illness [37]. A preclinical value was recorded retrospectively at visit 1. The score extends from 0 to 100. The Barthel-Index was first collected from the medical records, but due to invalid data, the Barthel-Index was later gathered by the research team. This led to a lower number of available Barthel-Index data (Table 3)
Early Rehabilitation Barthel Index	This extension of the Barthel-Index contains items like confusion, tracheostomy or dysphagia and is a valid and reliable tool for patients in neurological rehabilitation [38]. Score: − 325–0
Modified Medical Research Council dyspnea scale	This scale was used to evaluate dyspnea. The score ranges from 0 to 4, whereby 4 indicates the severest dyspnea [39]
Functional Ambulation Categories	The assessment extends from 0 to 5 and was used to classify walking ability [40]. Their good psychometric properties in neurological rehabilitation were shown [41] and the assessment was also used in patients with ICUAW [27]
Performance outcomes	
Grip strength	Grip strength was assessed twice on both hands with a digital dynamometer (Kern MAP 130K1, Balingen, Germany) and was measured in kilogram (kg). The maximum grip strength was standardized as a percentage of the reference grip strength, which was determined based on the patient's sex, age, and body height according to [42]
MRC sum score	Muscle strength was evaluated by manual muscle testing using the scoring system of the Medical Research Council (MRC). The scale ranges from 0 to 5, whereby 5 indicates normal muscle strength. The following functional muscle groups were evaluated: Shoulder abduction, elbow flexion, wrist extension, hip flexion, knee extension, ankle dorsal flexion [43, 44]. Consequentially, a maximum sum score of 60 is possible. A sum score of < 48 was repeatedly used as indicative for ICUAW [11, 13, 15]
Functional Status Score for the ICU	This scale measures basic physical functions on the ICU, comprises five items (e.g. rolling, transfer from supine to sitting) and has good psychometric properties [45]. Score: 0–35
Five Times Sit to Stand Test	This test was applied as it can be used to evaluate muscle strength of the lower extremities, risk of falling, dynamic balance and functional mobility [46–48] The test has good psychometric properties in patients after critical illness [49]
Box and Block Test	The Box and Block test [50] is a test for manual dexterity, where the patient is asked to transport as many blocks as possible from one compartment of a box to another within one minute. It shows a very high interrater- and test–retest-reliability and very good construct validity in neurologic patients [51]. Reference values were determined based on the patient’s sex and age [50]
Sensory examination	On the basis of the Fugl-Meyer-Assessment [52], the sensation of light touch (upper arm, the palmar surface of the hand, the thighs and plantar surfaces of the feet) and position of the joints (thumb (interphalangeal joint), wrist, elbow, gleno-humeral joint, ankle, knee, hip) was examined. Score 0–2, whereby 0 indicates anesthesia or absence of joint positions sense. Furthermore, vibration perception was evaluated with a vibrating tuning fork on the bony prominences ulnar styloid process, lateral epicondyle of the humerus, dorsum of the caput of os metatarsale I, the internal malleolus, and the tuberosity of the tibia. Values below 6/8 for the upper extremity and below 4/8 for the lower extremity indicated abnormal vibration perception [53]. Joint position sense and vibration examination were always started at the most distal body part and only continued to more proximal parts in case of pathological findings
Montreal Cognitive Assessment (MoCA)	The MoCA was used evaluate to cognitive function [54]. Good psychometric properties were so far only reported in patients with cerebrovascular diseases [55], but the MoCA was also already used in patients with critical illness and is recommended to evaluate cognitive function in post-intensive care syndrome [16, 56, 57]. Score 0–30, whereby a score < 26 indicated cognitive impairment

All assessments were conducted by experienced physiotherapists who were extensively trained in using the different outcome measures to minimize the risk of bias. Balance function and walking ability in this group of patients were analysed and described in [58].

### Patients’ characteristics and ICU data

Patients´ characteristics and data about the ICU stay were collected retrospectively using the electronic medical records and included the following parameters: age at disease onset, length of ICU stay, duration of mechanical ventilation, complications (e.g. sepsis, delirium), primary disease (type and duration), secondary diagnoses, duration of rehabilitation, and the Elixhauser Comorbidity Index [59]. Data about the preclinical status was collected during the first study visit and included the Functional Ambulation Categories, Clinical Frailty Scale, modified Rankin Scale, Barthel Index, consumption of alcohol and tobacco, physical activities, living conditions, relationship status, employment, and preclinical physical and cognitive disabilities.

Electrophysiological testing was used to confirm a potential diagnosis of CIP/CIM and was carried out shortly after study enrolment. The testing included motor and sensory nerve conduction velocities, compound muscle action potential after nerve stimulation (neCMAP) and after direct muscle stimulation (dmCMAP), and electromyography. More details and criteria used to diagnose CIP/CIM can be found in [26].

### Evaluation of PICS

In order to assess the percentage of patients with PICS at V1 and V2, we used assessments in accordance with a current Delphi study about instruments in PICS [57]. For physical function we used the MRC score, for cognitive function the MoCA, and for mental health the HADS. The following cut-offs were set to be indicative for impairments: Physical function impaired if MRC sum socre < 48 [13], mental health deficits if HADS > 7 separate for the two categories anxiety and depression [31], cognitive deficits if MoCA < 26 [54]. PICS was deemed to be present, if at least one of the three domains was impaired. Further aspects relevant to PICS, such as health-related quality of life (EQ-5D-5L), independence in activities of daily living (Barthel-Index), and pain, are not included in our PICS definition but were measured as part of the comprehensive assessment of health status (Table 1).

### Statistical analysis

Categorical variables are presented as absolute values and percentages, continuous variables as mean ± standard deviation or median (quartile 1-quartile 3).

#### Change in clinical outcome assessments

The Wilcoxon test was used to compare the two time points (V1 and V2) as data were either non-parametric or did not follow normal distribution (as checked by Shapiro–Wilk test and visually by means of QQ-plots). Effect sizes were calculated with r = z/√N. McNemar’s test was used for categorical values. Continuity Correction was used in case of < 30 discordant pairs. Cohen’s g (non-directional) was used to calculate the respective effect sizes [60].

#### Multiple logistic regression

Multiple logistic regressions were conducted to explore associated factors with (1) the rehabilitation outcome and (2) ICUAW at V2 (i.e., < 48 points according to the MRC sum score). The rehabilitation outcome was defined according to the modified Rankin Scale at V2, whereby the scores 0 to 2 indicated a desirable rehabilitation outcome and the scores 3 to 6 indicated a poor rehabilitation outcome.

Variables for the full model were included based on previous literature and expert knowledge. Independent variables for the rehabilitation outcome model included age, sex, obesity, MRC muscle sum score at V1, MoCA at V1, Elixhauser comorbidity index, duration of mechanical ventilation, diabetes, delirium, pre-existing mental health impairment, preclinical frailty (binary: 1–4 and 5–9), sepsis, ECMO, social support (approximated by the variable living alone vs. not alone), the primary diagnose acquired brain injury (stroke, hypoxia and traumatic brain injury), the primary diagnose COVID-19, and CIP/CIM (yes = CIP, CIM or CIP/CIM; no = no CIP/CIM). Independent variables for the ICUAW model included age, sex, obesity, grip strength (in % of reference), Elixhauser comorbidity index, duration of mechanical ventilation, diabetes, delirium, preclinical frailty, sepsis, ECMO, and CIP/CIM.

Variable selection was done according to the recommendations given by Heinze et al. [61]. The events-per-variable (EPV_global_) for the rehabilitation outcome model was 197/17 = 11.6 and for the ICUAW model 191/14 = 13.6. We conducted a backward elimination with the Akaike information criterion (AIC, significance level 0.157) as stopping criterion. Stability investigations of the selected model were performed according to Heinze et al. [61]. Bootstrap resampling with replacement (1,000 replicates) was done for the calculation of inclusion frequencies, sampling distributions of regression coefficients, and model selection frequencies. Furthermore, the relative conditional bias (which measures the anticipated level of bias introduced by variable selection when a particular independent variable is chosen) was calculated as suggested in Heinze et al. [61]. Postestimation shrinkage factors were calculated using the R package “shrink” [62]. The logistic regression model’s goodness-of-fit was assessed using a likelihood ratio test. We present the exponential of the coefficients as the odds ratio (OR) and their corresponding 95% confidence intervals. The Akaike Information Criterion (AIC) was reported for model comparison of the full and the selected model. Assumptions for the multiple logistic regression (linearity and influential values) were tested graphically for systematic violations in the selected model (Supplementary Figs. 1 and 2), whereby slight deviations were found. Multicollinearity was controlled by calculating variance inflation factors (VIF).

Statistical analyses were performed using R version 4.3.2. A p ≤ 0.05 (two-tailed) was considered significant. Missing data was not replaced.

## Results

**Fig. 1:**
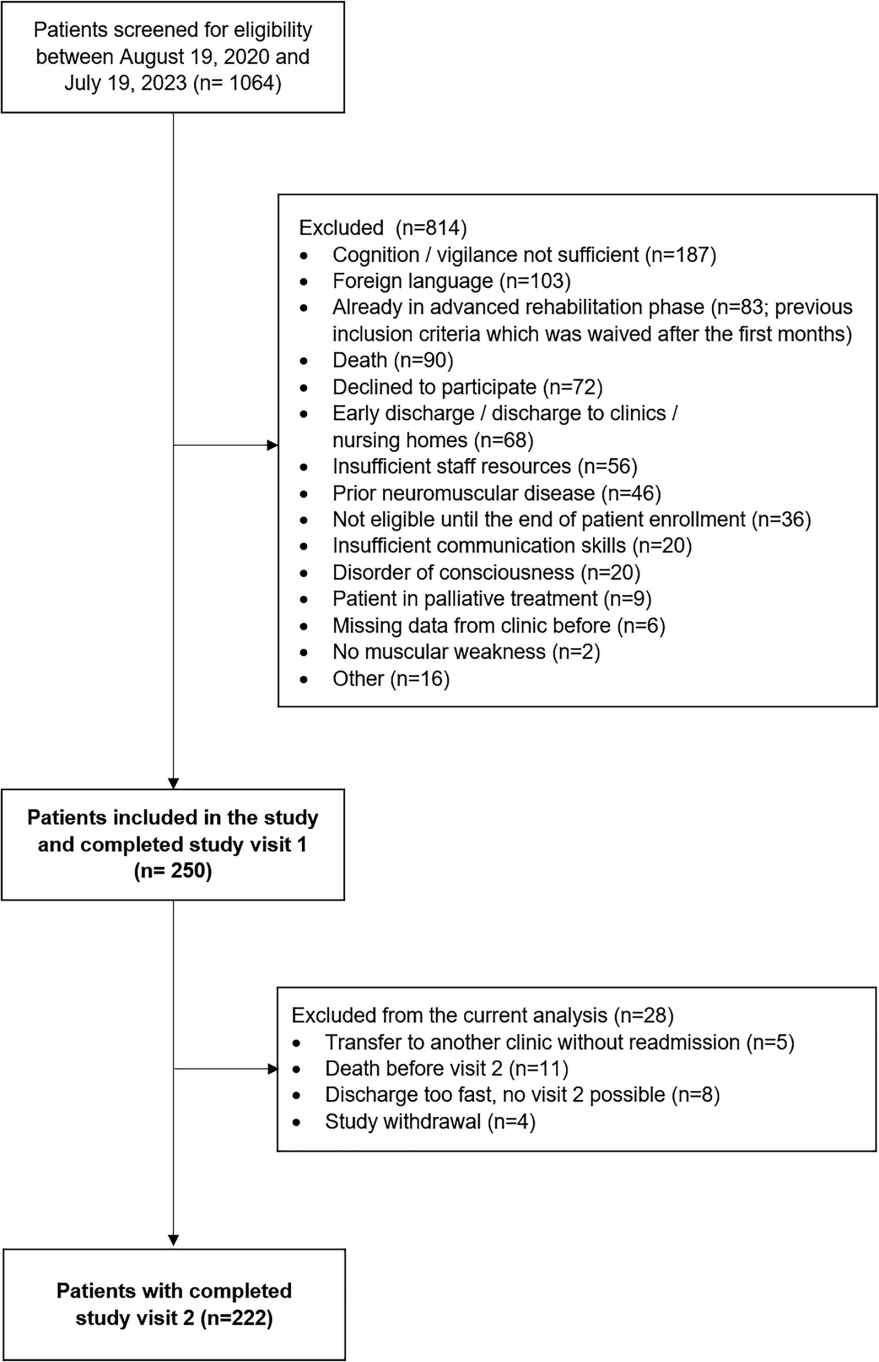
Flow Chart of the CINAMOPS study

We screened a total of 1064 patients and enrolled 250 patients between September 2020 and July 2023. The study visits (V1 and V2) were conducted between September 2020 and December 2023 (Fig. 1), whereby V1 was performed in 250 patients, V2 in 222 patients. Median time between the two study visits was 53 days. Eleven patients (4.4%) died after the first study visit. In five participants, the V2 was only conducted as a telephone interview. The characteristics of the study participants are presented in Table 2. In accordance with the extraordinary long median length of stay on ICU and duration of invasive ventilation, the diagnosis of chronic critical illness could be assigned to all patients (Table 2) [63]. The most frequent primary diagnoses among the included patients were COVID-19, cardiac diseases, and pulmonary diseases. Nerve conduction studies conducted in 216 patients revealed that 80.1% exhibited signs of CIP, CIM, or a combination of both.

**Table 2:** Demographic and clinical characteristics of critical illness survivors

Characteristics	Total population (n = 250)
Age, years	62.4 ± 13.6, min/max: 18/92
Sex, women	86 (34.4)
Length of hospitalization, days*	141 (98–192); 156.4 ± 81.8
Length of ICU stay, days	55 (39–78); 64.7 ± 40.3
Length of mechanical ventilation, days	39 (27–58); 45.8 ± 31.1
Length of neurological rehabilitation at Schoen Clinic Bad Aibling, days	67 (44–100); 82.2 ± 60.2
Time between	
First hospital admission and V1	81 (57–113); 90.8 ± 47.0
ICU discharge and V1	14 (8–23); 20.3 ± 18.4
V1 and V2	52 (29–82); 64.8 ± 50.8
Duration of ECMO (n = 51, 20.4%), days	10 (5–22.5); 17.6 ± 21.1
Primary diagnosis	
COVID-19	67 (26.8)
Cardiac disease	46 (18.4)
Pulmonary disease	45 (18.0)
Gastrointestinal/urological disease	25 (10.0)
Bacterial infection	21 (8.4)
Cerebral infarction / haemorrhage	20 (8.0)
Polytrauma	8 (3.2)
Oncological surgery	7 (2.8)
Hypoxia	5 (2.0)
Other	6 (2.4)
Chronic critical illness—conditions	
Prolonged acute mechanical ventilation (≥ 96 h)	250 (100.0)
Tracheotomy	209 (83.6)
Sepsis	149 (59.6)
Severe wounds	49 (19.6)
Stroke	33 (13.2)
Traumatic brain injury	6 (2.4)
Medical conditions	
Acute Respiratory Distress Syndrome (ARDS)	91 (36.3)
Sepsis	149 (59.6)
Acute kidney injury	134 (53.6)
Renal replacement therapy	88 (35.2)
Delirium	106 (42.4)
Severe encephalopathy	25 (10.0)
Multiple organ failure	23 (9.2)
Resuscitation	48 (19.2)
Cerebral ischemia	13 (5.2)
Nerve conduction studies^#^	
CIP	42 (19.4)
CIM	25 (11.6)
CIP/CIM	73 (33.8)
CIP but unclear CIM	22 (10.2)
No CIP but unclear CIM	11 (5.1)
No CIP/CIM	43 (19.9)
Comorbidities	
Diabetes (all type II)	47 (18.8)
Obesity	60 (24.0)
Hypertension	113 (45.2)
Psychiatric diagnose	42 (16.8)
Elixhauser Comorbidity Index	4.7 ± 7.0, min/max: -7/28
Preclinical status	
Frailty (Clinical Frailty Scale)	3 (2–3)
Disability (Modified Rankin Scale)	0 (0–1)
Barthel-Index	100 (100–100)
Occupation	
Employed	109 (43.6)
Retired	124 (49.6)
Student	3 (1.6)
Unemployed	14 (5.6)
Living conditions	
At home alone	52 (20.8)
At home not alone (e.g., with family)	194 (77.6)
Sheltered housing	2 (0.8)
Relationship	
Married/in a relationship	180 (72.0)
Single	41 (16.4)
Divorced	12 (4.8)
Widowed	17 (6.8)
Cigarette smoking	
Current smoker	43 (17.2)
Former smoker^##^	25 (10.0)
Non-smoker	182 (72.8)
Alcohol consumption	
Never	78 (31.2)
Once per month	35 (14.0)
2–4 times per month	37 (14.8)
2–3 times per week	29 (11.6)
4 times per week or more	73 (29.2)
Discharge destination	
Home	171 (71.6)
Further rehabilitation	29 (12.1)
Home with (mobile) nursing service	18 (7.5)
Nursing home	7 (2.9)
Outpatient intensive care unit	6 (2.5)
Sheltered housing	4 (1.7)
Other hospital	4 (1.7)
Death	11 (4.4)

Data are n (%), median (quartile 1-quartile 3) or mean ± SD

*ICU* intensive care unit, *ECMO* extracorporeal membrane oxygenation

^*^Hospitalization is defined as the period from the first day in hospital until discharge from rehabilitation

^#^Electrophysiological measurement was conducted in 216 persons (86.4%). Median time between disease onset and measurement was 91 days (67–127), the average time was 103.0 ± 50.5 days

^##^ Patients who quit smoking within the last 10 years

### Patient-reported outcomes

Patient-reported outcomes are displayed in Table 3. Health-related quality of life improved significantly during neurorehabilitation, as shown by large effect sizes. However, at discharge, the majority of patients faced problems with ambulation as well as activities of daily life and suffered from pain or discomfort. Fatigue, anxiety, and depression were observed to a lesser extent, with only about 20% of individuals exhibiting values above the respective cut-offs. Pain and sensory disturbances were reported by more than half of all participants and did not improve over the period of neurorehabilitation.

**Table 3:** Patient reported outcomes at admission to and at discharge from neurorehabilitation

	Visit 1 at study onset	Visit 2 at discharge	Test statistic	Effect size V1-V2
FSS-7	3.0 ± 1.6	2.7 ± 1.6	z = −2.79, p = 0.005	0.18
Fatigue ≥ 4	69 (28.4) n = 243	47 (21.7) n = 216	McNemar’s χ^2^ (1) = 2.04, p = 0.153	0.11
HADS				
Anxiety	5 (2–8)	3 (1–6.5)	z = −5.22, p < 0.001	0.35
Anxiety > 7	76 (30.9) n = 246	46 (21.2) n = 215	McNemar’s χ^2^ (1) = 7.52, p = 0.006	0.21
Depression	5 (3–9)	3 (2–6)	z = −6.55, p < 0.001	0.46
Depression > 7	89 (36.3) n = 245	39 (18.2) n = 214	McNemar’s χ^2^ (1) = 20.28, p < 0.001	0.31
EQ-5D-5L				
Visual Analogue Scale	48.9 ± 19.3 n = 248	61.2 ± 19.4 n = 217	z = −7.78, p < 0.001	0.54
Index value	0.39 ± 0.31 n = 248	0.64 ± 0.28 n = 228	z = −10.46, p < 0.001	0.70
Walking around	4 (3–5)	3 (2–3)	z = −9.60, p < 0.001	0.70
Problems with walking around	242 (98.0)	181 (83.4)	McNemar’s χ^2^ (1) = 29.12, p < .001	0.47
Washing/dressing	3 (2–4)	2 (1–2)	z = −9.92, p < 0.001	0.70
Problems with washing/dressing	218 (87.9)	128 (59.0)	McNemar’s χ^2^ (1) = 51.55, p < .001	0.41
Usual activity	4 (3–5)	2 (2–3)	z = −10.36, p < 0.001	0.73
Problems with usual activity	236 (95.1)	166 (76.5)	McNemar’s χ^2^ (1) = 35.28, p < 0.001	0.42
Pain or discomfort	3 (2–4)	2 (2–3)	z = −2.88, p = 0.004	0.20
Pain or discomfort is present	210 (84.7)	179 (82.5)	McNemar’s χ^2^ (1) = 0.28, p = 0.599	0.03
Anxiety or depression	2 (1–2)	1 (1–2)	z = −5.24, p < 0.001	0.35
Anxiety or depression is present	138 (55.6)	70 (32.3)	McNemar’s χ^2^ (1) = 37.7, p < 0.001	0.37
Presence of pain	142 (56.8) n = 250	126 (57.3) n = 220	McNemar’s χ^2^ (1) = 0.01, p = 0.906	0.01
Presence of sensory disturbances	141 (56.4) n = 250	135 (61.1) n = 220	McNemar’s χ^2^ (1) = 0.26, p = 0.612	0.03

Data are n (%), mean ± SD or median (quartile 1- quartile 3); FSS-7 = Fatigue-Severity-Scale-7; HADS = Hospital Anxiety and Depression Scale; EQ-5D-5 l = EuroQol—5 dimensions—5 level; The effect sizes (of the Wilcoxon tests) were calculated with $r=z/\sqrt{N}$. Effect sizes are small (≥ 0.1), moderate (≥ 0.3) or large (≥ 0.5) according to Cohen (1988), p.79–81 [60]. Effect sizes for McNemar´s tests were calculated with the non-directional Cohen`s g are interpreted as small (0.05 to < 0.15), medium (0.15 to < 0.25), and large (≥ 0.25) according to Jacob Cohen: Statistical Power Analysis for the Behavioral Sciences (1988), p.147–149 [60]

### Clinician-reported outcomes

Clinician-reported outcomes are reported in Table 4. All parameters improved significantly during neurologic rehabilitation, and patients improved substantially as displayed by the large effect sizes. However, the preclinical status as indicated by the preclinical medians (Table 2) of the Barthel Index, the modified Rankin Scale, and the Clinical Frailty Scale could not be regained. According to the outcome categories, the median patients at discharge exhibited mild frailty, moderate disability, and experienced shortness of breath when hurrying or walking up a slight incline.

**Table 4:** Clinician-reported outcomes at admission to and at discharge from neurorehabilitation

	Visit 1 at study onset	Visit 2 at discharge	Wilcoxon test statistic	Effect size V1-V2
Modified Rankin Scale	4 (4–5) n = 248	3 (2–4) n = 233	z = −11.06, p < 0.001	0.75
Clinical Frailty Scale	7 (6–7) n = 248	5 (4–6) n = 222	z = −11.29, p < 0.001	0.80
Barthel-Index	45 (20–75) n = 136	85 (70–100) n = 132	z = −9.02, p < 0.001	0.85
Early Rehabilitation Barthel Index	−50 (−100; 0) n = 250	0 (0–0) n = 236	z = −9.56, p < 0.001	0.69
Functional Ambulation Categories	2 (0–3) n = 250	4 (3–5) n = 222	z = −11.64, p < 0.001	0.83
Modified Medical Research Council Dyspnea Scale	3 (1–4) n = 208	1 (0–2) n = 197	z = −7.28, p < 0.001	0.55

Data are displayed as median (quartile 1- quartile 3); The effect size was calculated with $r=z/\sqrt{N}$. Effect sizes are small (≥ 0.1), moderate (≥ 0.3) or large (≥ 0.5) according to Cohen (1988), p.79–81 [60]

### Performance outcomes

Performance outcomes are displayed in Table 5. All motor performance outcomes improved significantly with large effect sizes. However, muscle strength was still substantially reduced at discharge, as the maximum handgrip strength was on average only 52% of the respective reference values. The cognitive function also showed significant improvement, albeit with a small effect size. The somatosensory investigation revealed more severe deficits in the lower extremity. Around 25% of individuals experienced deficits in the superficial sensation of the lower extremity, which did not improve over time. Vibration perception of the lower extremity was impaired in 50% at V1 and improved significantly over time; however, 41% still had deficits at V2. Impairments in proprioception occurred only in the minority of patients.

**Table 5:** Performance outcomes at admission to and at discharge from neurorehabilitation

	Visit 1 at study onset	Visit 2 at discharge	Test statistic	Effect size V1-V2
Maximum grip strength in kg	15.5 ± 7.5	20.5 ± 7.6	z = −11.20, p < 0.001	0.77
Maximum grip strength in % of reference	39.3 ± 17.6 n = 247	51.8 ± 16.2 n = 216	z = −11.22, p < 0.001	0.77
MRC Sum Score	40 (35–44)	45 (41–48.5)	z = −10.96, p < 0.001	0.76
MRC Sum Score < 48	216 (86.8) n = 249	141 (65.6) n = 215	McNemar’s χ^2^ (1) = 40.69, p < 0.001	0.44
Functional Status Score for the ICU	27 (17–32) n = 249	34 (32–35) n = 221	z = −11.56, p < 0.001	0.81
Five Times Sit to Stand Test	20.0 ± 10.0	16.9 ± 7.4	z = −7.19, p < 0.001	0.67
Help required	91 (70.5)	122 (63.9)		
Not possible	114 (46.9) n = 129	19 (9.0) n = 191		
Box and Block Test				
Right	45.4 ± 17.6	55.8 ± 17.1	z = −9.34, p < 0.001	0.69
Right in % of reference	66.1 ± 20.2	79.4 ± 20.2	z = −9.64, p < 0.001	0.73
Left	42.3 ± 18.5	51.8 ± 18.5	z = −9.26, p < 0.001	0.69
Left in % of reference	64.4 ± 20.7 n = 223	78.2 ± 18.6 n = 203	z = −9.49, p < 0.001	0.73
MoCA	23.7 ± 4.3	24.4 ± 4.1	z = −2.08, p = 0.038	0.15
MoCA < 26	154 (64.7) n = 238	116 (55.2) n = 210	McNemar’s χ^2^ (1) = 4.19, p = 0.041	0.12
Deficits in superficial sensation (light touch)				
- Upper extremity	44 (17.7)	28 (13.2)	McNemar’s χ^2^ (1) = 2.94, p = 0.086	0.15
- Lower extremity	68 (27.4) n = 247	55 (25.9) n = 212	McNemar’s χ^2^ (1) = 0.22, p = 0.639	0.04
Deficits in proprioception awareness				
- Upper extremity	18 (7.3)	10 (4.7)	McNemar’s χ^2^ (1) = 4.08, p = 0.043	0.33
- Lower extremity	19 (7.7) n = 247	12 (5.7) n = 212	McNemar’s χ^2^ (1) = 0.64, p = 0.423	0.14
Deficits in vibration perception				
- Upper extremity	26 (10.5)	21 (10.2)	McNemar’s χ^2^ (1) = 0.19, p = 0.663	0.07
- Lower extremity	124 (50.2) n = 247	85 (41.3) n = 212	McNemar’s χ^2^ (1) = 4.41, p = 0.035	0.15

Data are n (%), mean ± SD or median (quartile 1- quartile 3); MoCA = Montreal Cognitive Assessment; The effect sizes (of the Wilcoxon tests) were calculated with $r=z/\sqrt{N}$. Effect sizes are small (≥ 0.1), moderate (≥ 0.3) or large (≥ 0.5) according to Cohen (1988), p.79–81 [60]. Effect sizes for McNemar´s tests were calculated with the non-directional Cohen`s g are interpreted as small (0.05 to < 0.15), medium (0.15 to < 0.25), and large (≥ 0.25) according to Jacob Cohen: Statistical Power Analysis for the Behavioral Sciences (1988), p.147–149 [60]

### PICS evaluation

**Fig. 2:**
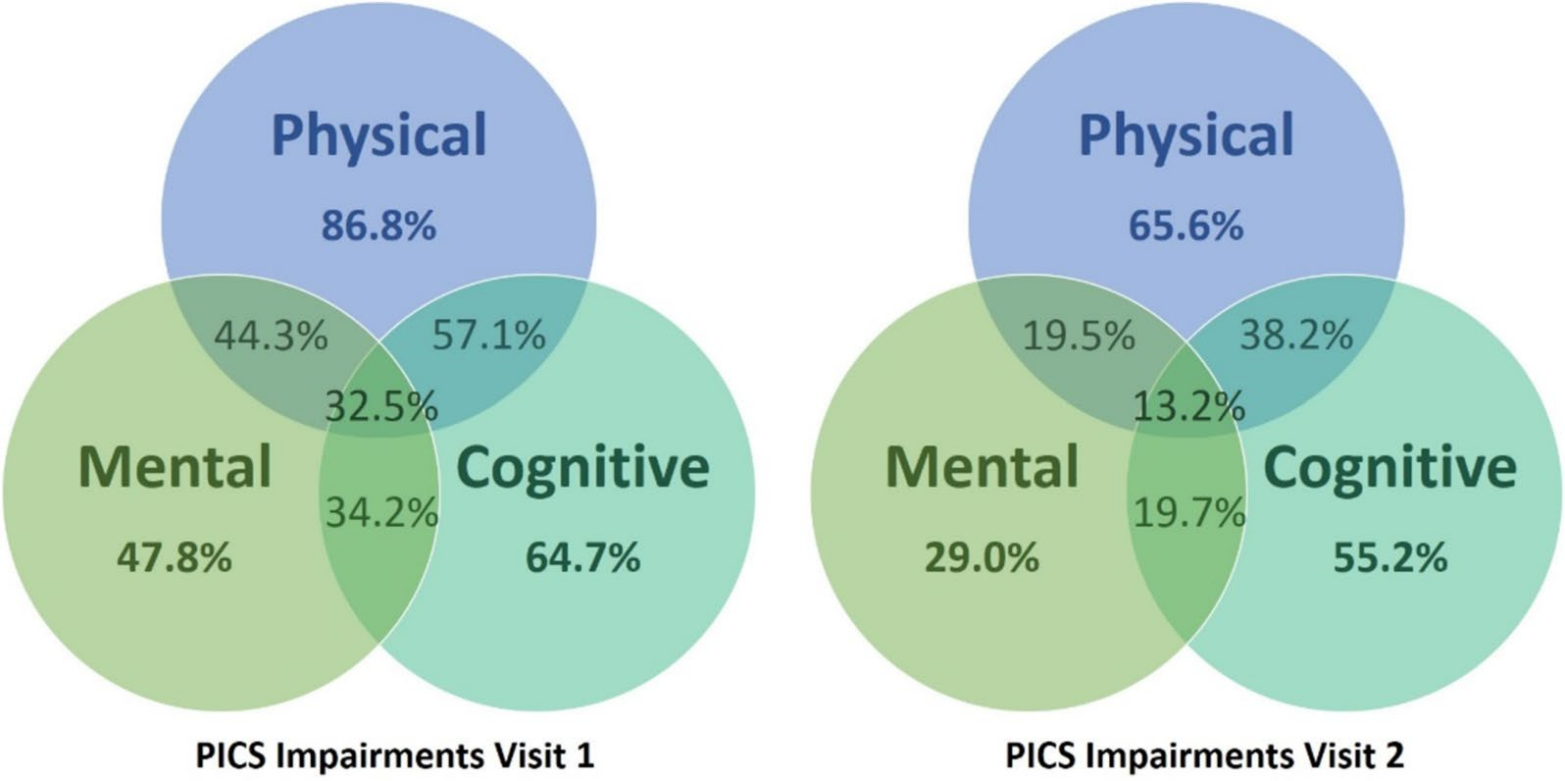
PICS impairments in each domain and their overlap at study visits 1 and 2

Figure 2 shows impairments according to PICS at V1 and V2 in each domain (physical, mental and cognitive impairments) and the overlap of the domains. At V1, 239 patients (95.6%) and at V2 still 185 (84.5%) suffered from PICS. At both time points, participants were mainly affected by physical symptoms (216 (86.8%) at V1 and 141 (65.6%) at V2). Cognitive symptoms were more frequent than mental symptoms at both study visits. PICS burden decreased in all domains over the period of neurorehabilitation. However, physical and cognitive impairments were still substantial at discharge from rehabilitation.

### Logistic regression for poor rehabilitation outcome

**Fig. 3:**
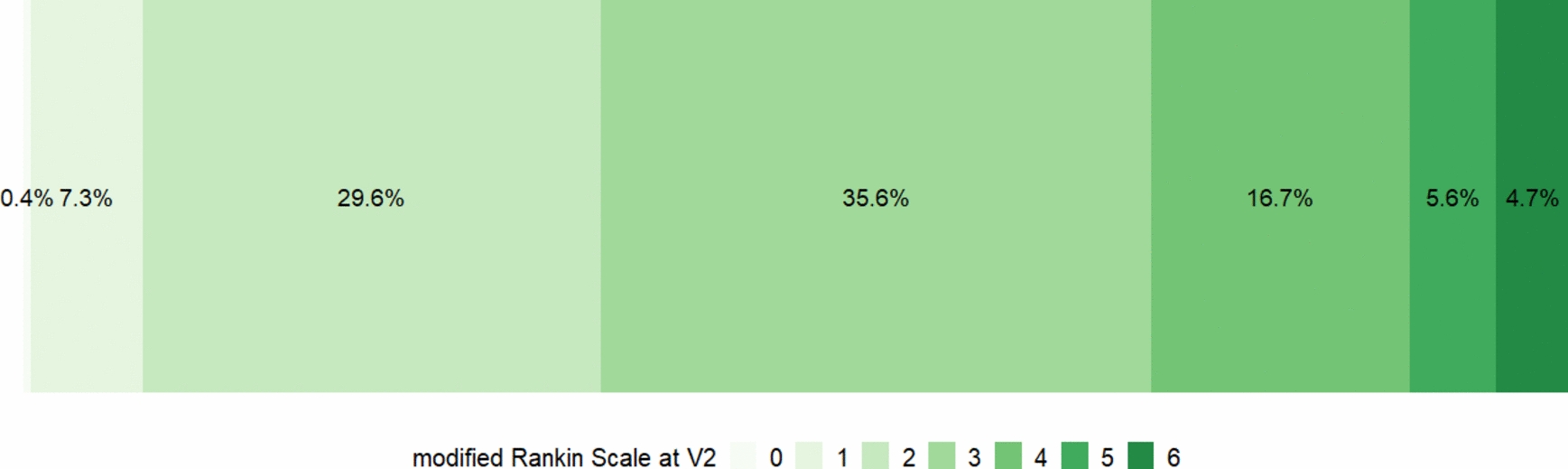
Distribution of the modified Rankin Scale at V2

The distribution of the modified Rankin Scale for measuring the rehabilitation success is shown in Fig. 3. A poor rehabilitation outcome as reflected by the modified Rankin Scale scores 3 to 6 was seen in 63% of the individuals.

The multiple logistic regression model for the rehabilitation outcome included the variables muscle strength, duration of mechanical ventilation, acquired brain injury, sex, age, diabetes and the Elixhauser comorbidity index after backward elimination (Table 6). Only muscle strength, duration of mechanical ventilation, and sex were significantly associated with the rehabilitation outcome (p < 0.035). Acquired brain injury was on the border to significance (p = 0.059). Each additional point in the MRC sum score at V1 decreased the chance of a poor rehabilitation outcome by 15%. Each additional day of mechanical ventilation increased the chance of a poor rehabilitation outcome by 2%. The chance of a poor rehabilitation outcome in females is (1/0.45 =)2.22-times higher than in males. The bootstrapping results, model selection frequencies, and parameterwise shrinkage factors are presented in Supplementary Tables 1, 2, and 5, along with an interpretation of the results. In short, according to the model stability investigations, the associations between the rehabilitation outcome and muscle strength, duration of mechanical ventilation, sex, and potentially acquired brain injury seem plausible. In contrast, diabetes, the Elixhauser comorbidity index, and age are less certain as indicated by lower bootstrap inclusion frequencies, contradictory model selection frequencies, and low shrinkage factors.

**Table 6:** Results of the multiple logistic regression analyses

Model poor rehabilitation outcome			Model ICUAW		
	Odds Ratio	95% CI		Odds Ratio	95% CI
Intercept	173.98	9.06; 4246.63	Intercept	2.95	0.39; 23.67
**MRC Sum V1**	**0.85**	**0.79; 0.90**	**Grip strength % V1**	**0.93**	**0.91; 0.96**
**Duration MV**	**1.02**	**1.005; 1.03**	**Age**	**1.04**	**1.02; 1.08**
**Sex**			**Sex**		
**Women**	**Reference**		**Women**	**Reference**	
**Men**	**0.45**	**0.21; 0.93**	**Men**	**0.42**	**0.19; 0.89**
Brain injury			**Elixhauser**	**1.06**	**1.01; 1.13**
No	Reference				
Yes	3.22	1.00; 11.61			
Diabetes			Obesity		
No	Reference		No	Reference	
Yes	2.23	0.91; 5.85	Yes	2.38	1.01; 5.88
Elixhauser	1.04	0.99; 1.10	ECMO		
			No	Reference	
			Yes	0.42	0.17; 1.06
Age	1.02	0.99; 1.05	Brain injury		
			No	Reference	
			Yes	2.90	0.90; 10.35
R^2^ (Nagelkerke)	0.360			0.331	
Somer’s D	0.601			0.585	
Chi^2^-Statistic	60.60 (df = 7); p < .001			52.01 (df = 7); p < .001	

Significant values are in bold. *ICUAW* Intensive care unit-acquired weakness, *MV* mechanical ventilation, *ECMO* Extracorporeal membrane oxygenation

### Logistic regression for ICUAW at discharge

The selected model of the multiple logistic regression for ICUAW at discharge contained the variables handgrip strength, age, sex, Elixhauser comorbidity index, obesity, ECMO, and acquired brain injury after backward elimination. Only handgrip strength, age, and Elixhauser reached statistical significance (p < 0.036). Each additional percentage point in the handgrip strength decreased the chance of ICUAW by 7%. Every additional year of age increased the chance of ICUAW by 4%. The odds of ICUAW among male patients was 0.42-times the odds for female patients. This means that the chance of ICUAW among females was (1/0.42 =)2.38-times higher than for males. Each additional point in the Elixhauser comorbidity index increased the chance of ICUAW by 6%. Bootstrapping results, model frequencies, and parameterwise shrinkage factors are presented in Supplementary Tables 3, 4, and 5, along with an interpretation. In brief, the model stability investigation supports the final selected model, although some uncertainty exist for the non-significant values as displayed by the higher bias percentages and lower shrinkage factors.

## Discussion

Here we describe the health status of critical illness survivors at the beginning and the end of neurological rehabilitation. Outcomes show the overall good improvement during rehabilitation. Nonetheless, a majority of individuals exhibited ongoing impairments upon discharge, with PICS prevalent in 84.5% of individuals, alongside high rates of physical and cognitive impairments. The preclinical health status was mostly not yet regained. We found that muscle strength, duration of mechanical ventilation, and female sex were associated with a poor rehabilitation outcome. Furthermore, we found higher age, lower handgrip strength, female sex, and more comorbidities to be associated with higher odds of ICUAW at discharge.

### Outcome after rehabilitation in survivors of critical illness

Studies in survivors of critical illness describing the course of rehabilitation or including comprehensive outcomes are sparse. There are some studies which reported significant improvements after inpatient rehabilitation, but the description of outcomes is often narrowed down to the Barthel-Index, modified Rankin Scale or Functional Independence Measure (FIM) [64–66]. One study with a set of comprehensive assessments described an 8 week course at a post-ICU hospital and inpatient rehabilitation of patients with ICUAW [27]. All outcome parameters of physical and cognitive function, except pain, improved significantly until discharge, but, the health status at discharge was substantially more impaired compared to our cohort. A shorter time since disease onset and a longer duration of mechanical ventilation in this study may have contributed to these differing findings. Another study reported the outcome of CIP/CIM patients after an average of 11 weeks of rehabilitation [67]. The MRC sum score, the modified Rankin Scale, and the Barthel-Index improved over time and the scores were similar to those we reported. In consequence of our results and the results of prior studies, inpatient rehabilitation seems beneficial for improving the physical functioning of survivors of critical illness. Our study also suggests a slight positive influence on cognitive and mental health, which requires further evaluation. However, randomized controlled trials are needed to evaluate the true effect of rehabilitation and to investigate therapies with the greatest positive influence.

We reported a comprehensive set of outcome assessments. As hardly any psychometric properties have been evaluated for critical illness survivors thus far, we demonstrated their suitability for this patient group and provided reference values. We successfully conducted all performance outcomes except the Five Times Sit to Stand test, which only 129 patients could complete at V1 shortly after ICU discharge. Future studies should investigate psychometric properties of assessment tools for their use in patients after critical illness, as our group recently did by evaluating the Mini-BESTest for assessing balance [58].

Investigations of chronically critically ill patients are rare. It was reported that 65% had a devastating outcome at 1 year with complete functional dependency and death [68] and that only around 20% will return home [63]. Our cohort showed superior health statuses, as only 10.3% had a very poor outcome (modified Rankin Scale 5 and 6) and 72% were discharged home. Further studies with chronically critically patients are needed to contextualize our results.

### Frequencies of PICS and related aspects

In critically ill patients after COVID-19, 90–94% met the criteria for PICS at 1.5–3 months after ICU discharge [69–71]. Our PICS frequencies are therefore in line with previous results. Reported frequencies in the domains varied, which is also due to differences in diagnosing. Physical impairment was reported in 81–87% [69, 71], which is slightly higher than our results. However, they measured physical impairment with the EQ-5D-5L, in which we found similar percentages of problems in the domains walking, and pain and discomfort. Cognitive impairment varied from 25 to 67% [69, 71, 72] and mental impairment was reported in 49% [69, 71], which was substantially more frequent compared to our cohort. However, PICS is more than just ICUAW and concerning values in HADS or MoCA. Thus, our definition is a simplification of PICS and there might be patients with impairments in activities of daily living, sleep, health-related quality of life or with post-traumatic stress disorder, which we have not considered. Furthermore, it was recently suggested that chronic pain be included in PICS as it was frequently (up to 77%) reported in patients following ICU discharge [73]. In our cohort, 57% of individuals reported pain, which did not improve during neurorehabilitation. This topic requires further attention, as pain can become chronic, greatly affects daily life and often correlates with both anxiety and depression [74].

Fatigue was reported in 47% of chronically critically ill patients at 3 months [75] and in 70% of ARDS survivors at 6 months [76]. Frequency was also high in critical illness survivors due to COVID-19, especially 3–12 months after discharge from rehabilitation (45–55%) [77]. Consequently, while not desirable, we anticipate an increase in the relatively low fatigue frequency for this cohort over the long-term, consistent with an increase in anxiety and depression as recently reported [77].

Health-related quality of life [78] improved during neurorehabilitation but was still substantially reduced at discharge compared to a German general population of equal age [79] and compared to critical COVID-19 survivors [3, 80].

In short, the high frequency of PICS impairments and associated symptoms underscores their clinical and scientific importance. However, our definition of PICS likely simplifies the true and more comprehensive impairments experienced by affected individuals, which needs to be considered in future studies.

### Associated factors with rehabilitation outcome and persistent ICUAW

Factors associated with the rehabilitation outcome after critical illness have scarcely been investigated thus far. Miyamoto et al. [81] reported older age, more than one preclinical comorbidity and longer duration of mechanical ventilation as being associated with a worsened status in activities of daily living 3 months after ICU discharge [81]. Kang and Lee 2024 investigated physical impairment in activities of daily living 3 months after ICU discharge and reported female gender, preclinical comorbidities, and a longer ICU stay as associated with physical impairments [82]. According to a meta-analysis, high disease severity, older age and female sex were significant risk factors for physical impairment irrespective of their occurrence date [5]. These results are in line with our results, where longer duration of mechanical ventilation, female sex, and more comorbidities were also associated with a poor rehabilitation outcome.

Factors associated with the occurrence of ICUAW beyond the ICU have scarcely been investigated. Benedini et al. [83] found a strong correlation between handgrip strength and long-term muscle weakness of the lower extremities, which corresponds to our results. Higher age and female sex were previously associated with ICUAW at ICU, which is in line with our results for persistent ICUAW [84]. Contrary to previous findings, the duration of mechanical ventilation was not associated with persistent ICUAW, a factor often deemed significant in previous reports on ICUAW risk within the ICU [13, 84]. ECMO was included in the model despite lacking statistical significance, and the OR indicated a lower risk of ICUAW in ECMO-treated patients, contrary to expectations [85]. Notably, ECMO patients in our cohort were significantly younger than non-ECMO patients, which may explain this unexpected finding.

Although we expected CIP/CIM and preclinical frailty to be associated with the rehabilitation outcome [86, 87], this was not supported by our analyses. Acquired brain injury was included in both models, reinforcing prior findings that critically ill stroke patients, especially those requiring mechanical ventilation or with CIP/CIM, have poor outcomes [88–90].

### Practical implications

Although significant improvements were found after neurorehabilitation, most patients did not regain their preclinical health status. PICS was still highly prevalent at discharge from rehabilitation. Therefore, follow-up investigations and ongoing intensive and tailored therapies, implemented for example within a PICS follow-up system [91], are highly recommended. The results of the regression analyses provide insights into the relationships between variables and the likelihood of poor rehabilitation outcomes and persistent ICUAW. As female gender, age, and the Elixhauser comorbidity index were included in the final regression models, women and older individuals with more comorbidities should receive special attention in the long term. Duration of mechanical ventilation was associated with a poor rehabilitation outcome, suggesting prolonged ventilation might impact recovery, though causality remains unclear. The strong association between muscle strength post-ICU discharge and rehabilitation outcomes indicates that interventions aiming to enhance muscle strength could be beneficial, but further research is needed to determine their effectiveness.

### Strengths and limitations

Strengths of this study are the comprehensive set of outcome measures, the large number of enrolled patients, and the electrophysiological testing of CIP/CIM. A limitation for generalization of our results might be the high severity of critical illness in our participants, who had extraordinarily long durations of ICU treatment and mechanical ventilation, with all patients fulfilling the criteria for chronic critical illness. Although the results might therefore not be generalizable for all critical illness survivors, it is important to report these patients. Furthermore, as patients were only included in the study when communication abilities were sufficient to perform the assessments, patients with disorders of consciousness, severe dementia, and severe aphasia were not included. Therefore, future studies should consider the inclusion of non-communicative individuals with an adapted study design.

Another limitation is the lack of established psychometric properties for most of the outcome measures. While these measures are frequently used in critical care research and recommended for assessing PICS, the psychometric properties have been insufficiently evaluated. Future studies are required to assess parameters such as reliability and validity, to ensure the robustness and accuracy of the measurements.

As we did not include a control group receiving another type of intervention, no conclusions can be made about the causal effect of neurorehabilitation approaches in critical care survivors. Thus, randomized controlled trials are needed to identify the most effective treatments for improving the outcome of critical illness survivors and patients with PICS/ICUAW [19, 20].

The results of the logistic regressions should be interpreted with caution. Variable selection is widely debated, as it often introduces uncertainty and can impair the validity of results [92]. To address this issue, we followed the recommendations by Heinze et al. [61], conducted the preferred variable selection procedure backward elimination with AIC as stopping criterion (instead of using pre-specified significance levels), and applied post-estimation shrinkage methods and model stability investigations. However, we did not perform external validation, which could have provided further insights into the model’s robustness and generalizability. The potential for overfitting should also be considered.

The exclusion criterion of no muscular weakness (i.e., MRC 5/5) could be discussed. It was chosen to ensure the inclusion of only patients showing signs of functional impairment; however, it does not align with typical characterizations like ICUAW. Since only 2 out of 814 patients were excluded due to this criterion, it did not result in a different subset of critical illness survivors than those usually treated at our hospital.

## Conclusions

In this prospective cohort study with survivors of critical illness, we demonstrated significant improvements during rehabilitation across a comprehensive set of patient-reported, clinician-reported and performance outcomes. Despite the positive progress, PICS was still present in 85% of individuals at discharge from rehabilitation, whereby mostly physical and cognitive impairments persisted. Accordingly, health-related quality of life was substantially reduced. We found that lower muscle strength after ICU discharge, female gender, longer duration of mechanical ventilation, higher age, and more comorbidities were associated with poor rehabilitation outcomes. Therefore, follow-up investigations and ongoing intensive, tailored, and multidisciplinary therapies are indicated in survivors of critical illness. Accordingly, randomized controlled trials are required to identify the most effective treatments.

## Supplementary Information

Supplementary material 1

## Data Availability

The datasets used and/or analysed during the current study are available from the corresponding author on reasonable request.
