# Supplementary material for: Comprehensive assessment and progression of health status during neurorehabilitation in survivors of critical illness: a prospective cohort study
Source: Ann Intensive Care. 2024 Nov 26;14:175. doi: 10.1186/s13613-024-01396-x (PMC11599680; doi:10.1186/s13613-024-01396-x)
Supplement: Supplementary file 1 — Supplementary material 1 [file 13613_2024_1396_MOESM1_ESM.pdf]

Comprehensive assessment and progression of health status during neurorehabilitation in survivors of critical illness: a prospective cohort study

Marion Egger, Melanie Finsterhölzl, Daria Farabegoli, Franziska Wippenbeck, Maria Schlutt, Friedemann Müller, Volker Huge, Klaus Jahn, Jeannine Bergmann

Corresponding author: Marion Egger (megger@schoen-klinik.de)

Supplementary Figure 1 Linearity assumption of continuous predictors and the logit of the outcome for model of rehabilitation outcome (modified Rankin Scale)

MRC Sum Score V1

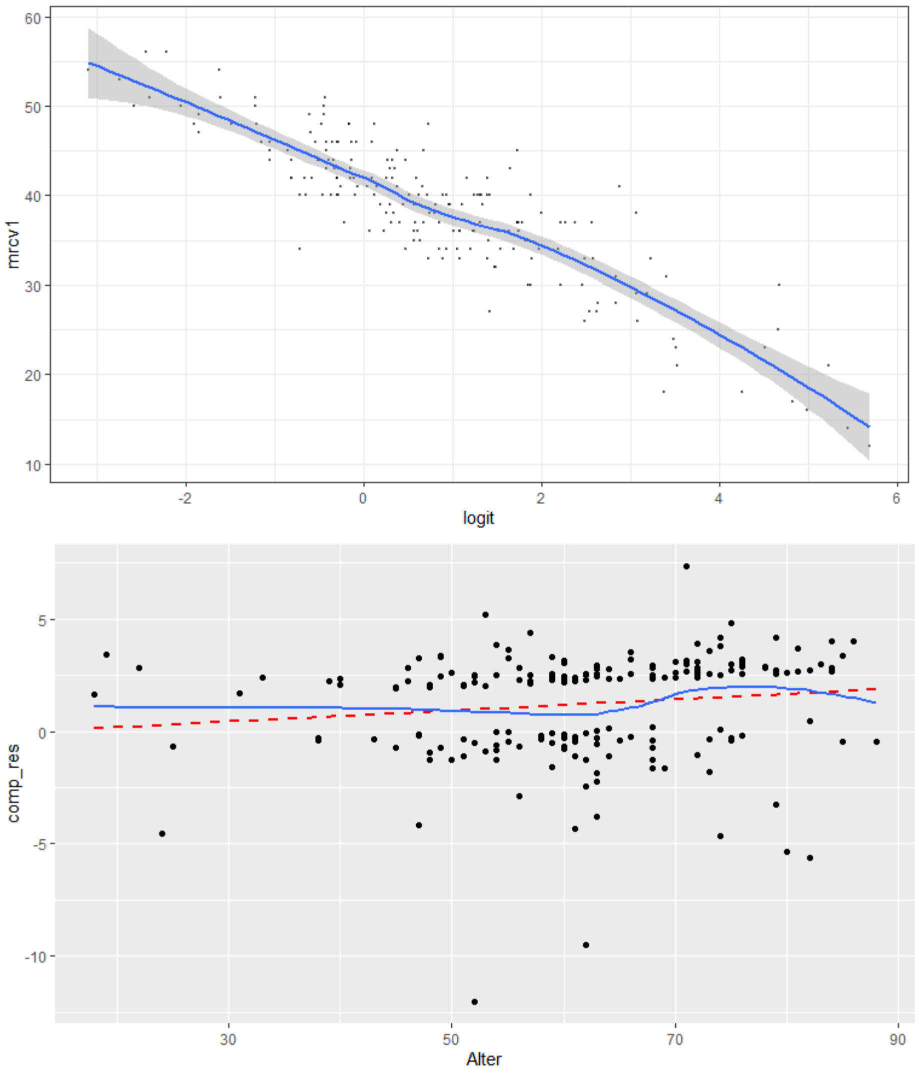

Elixhauser Comorbidity Scale	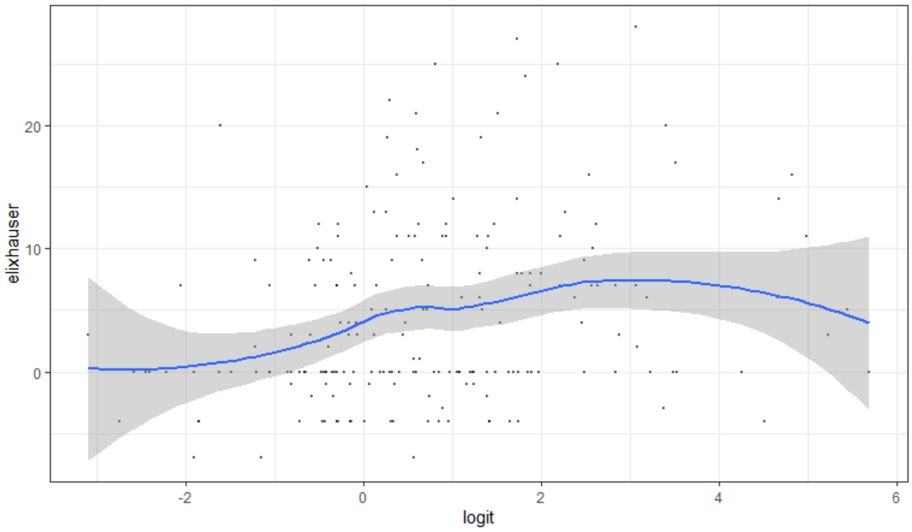 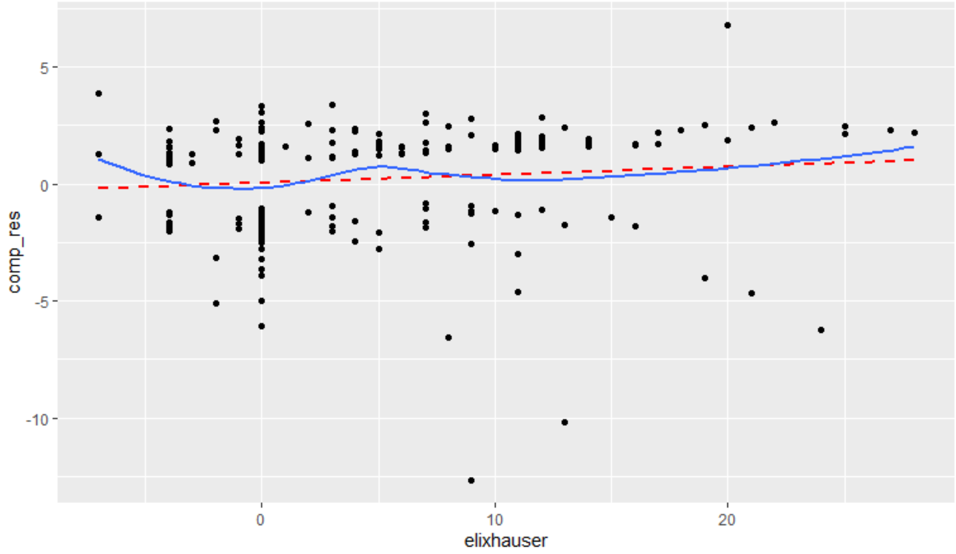
Duration of mechanical ventilation	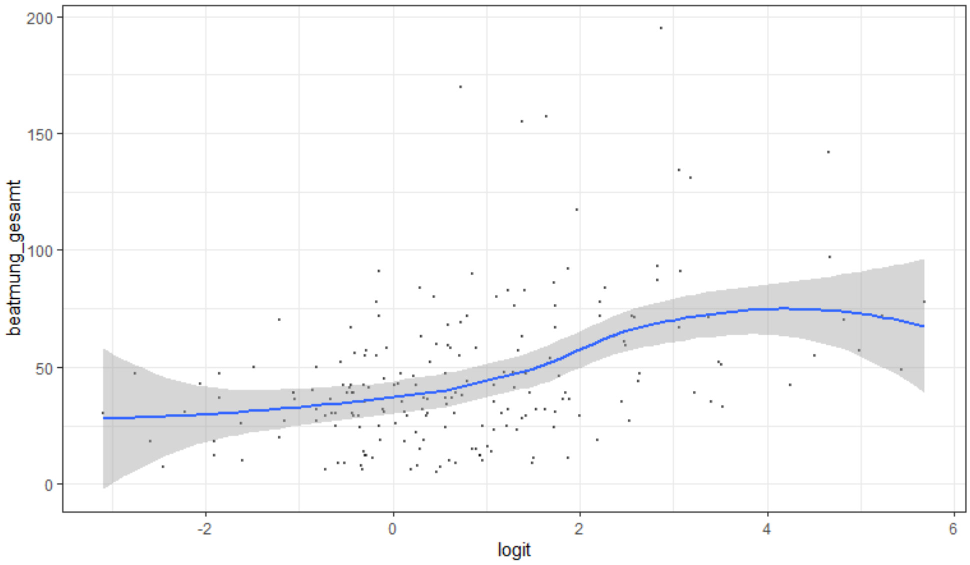

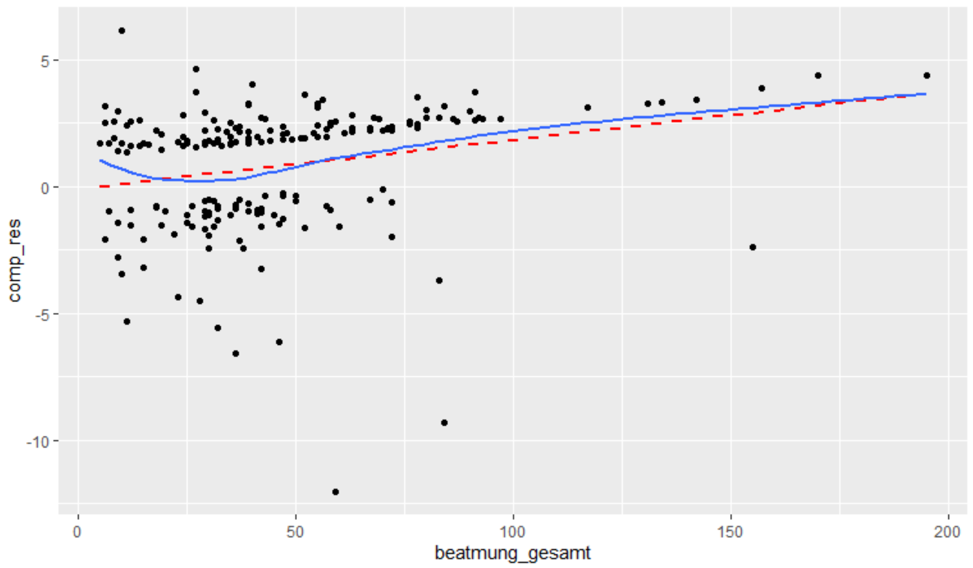

comp_res
5
0
-5
-10
0
50
100
150
200
beatmung_gesamt

Age

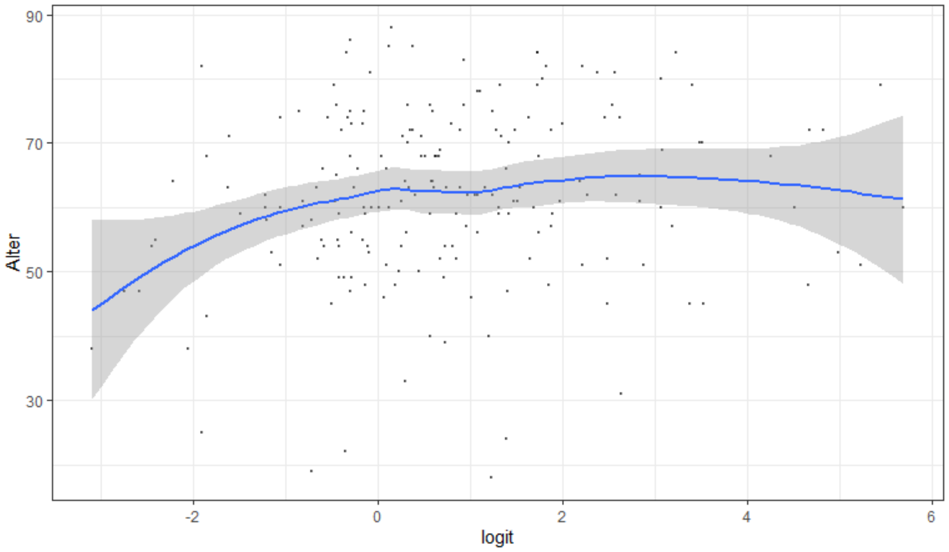

Alter
90
70
50
30
-2
0
2
4
6
logit

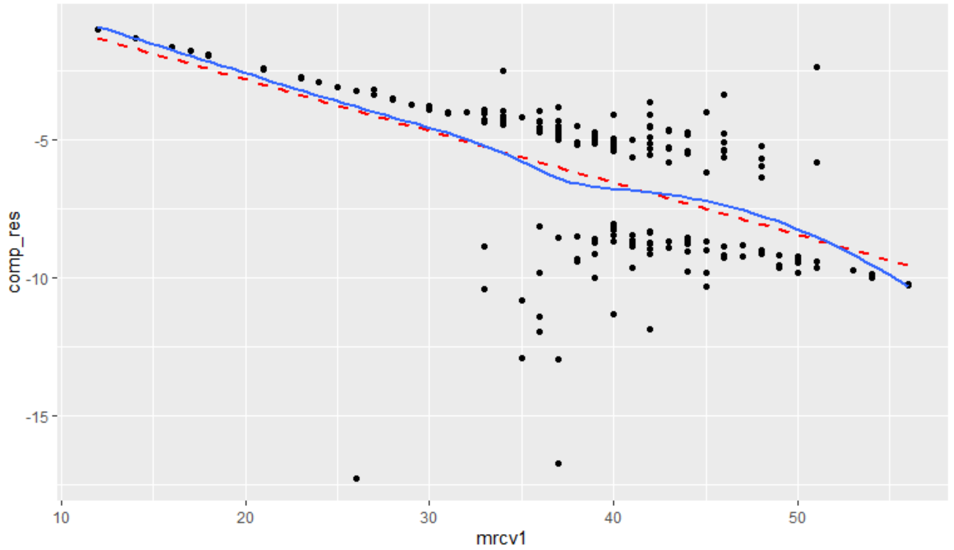

comp_res
-5
-10
-15
10
20
30
40
50
mrcv1

Supplementary Figure 2 Linearity assumption of continuous predictors and the logit of the outcome for model of muscle weakness outcome (MRC sum score)

Handgrip strength at V1	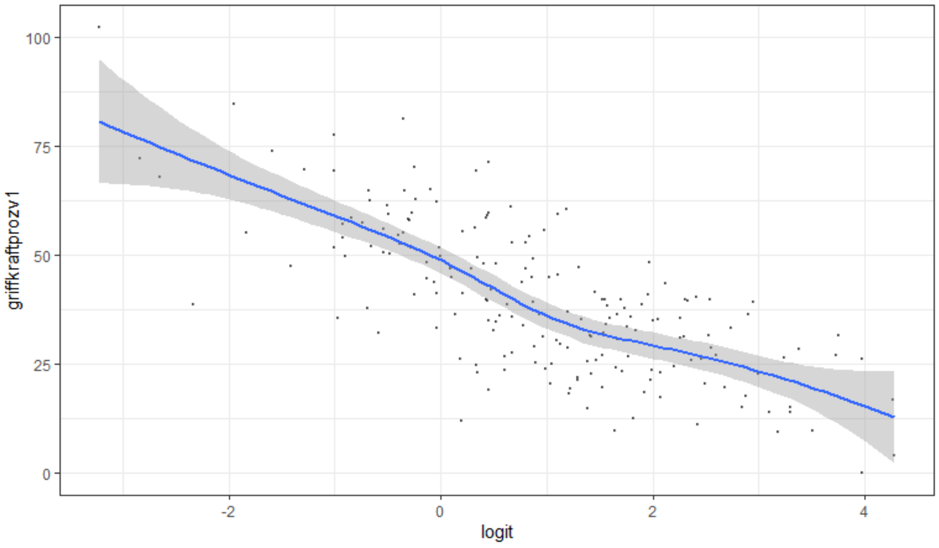 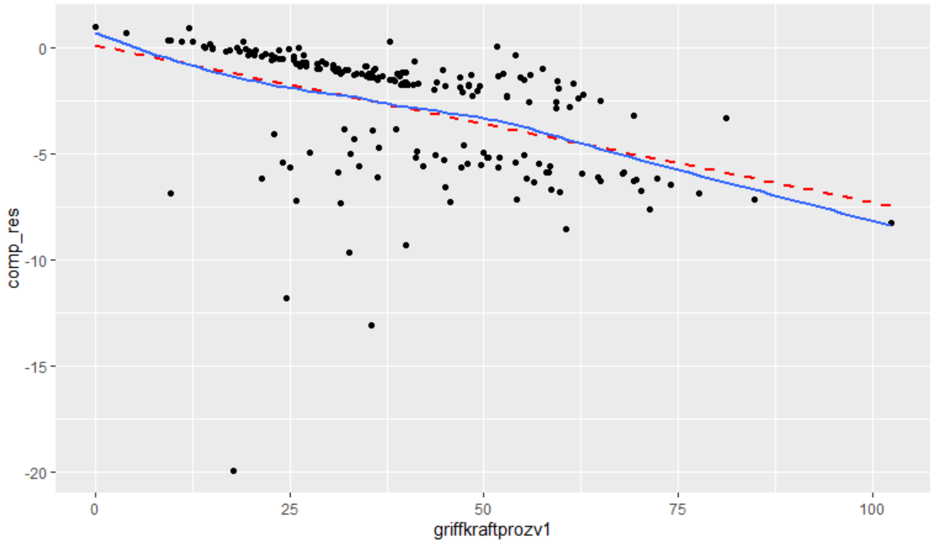
Elixhauser Comorbidity Scale	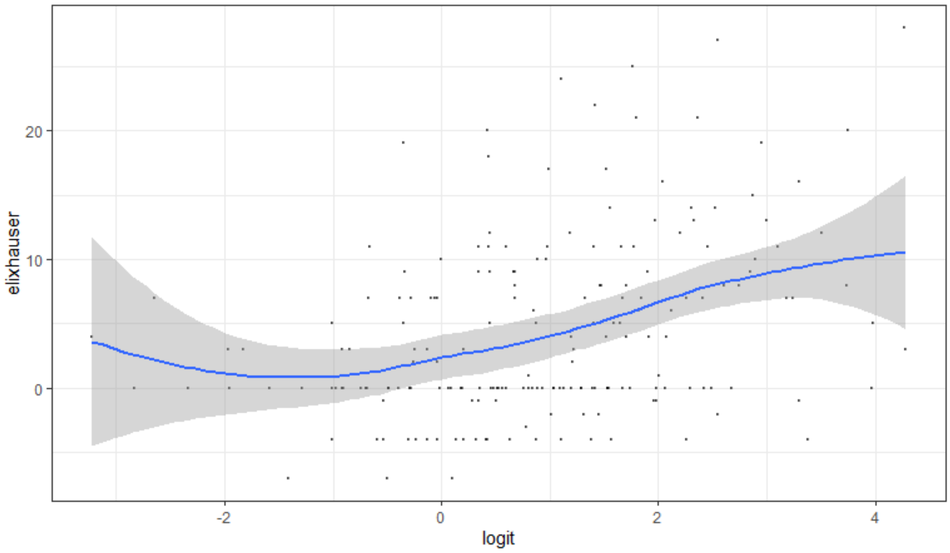

<table>
<tr><td></td><td>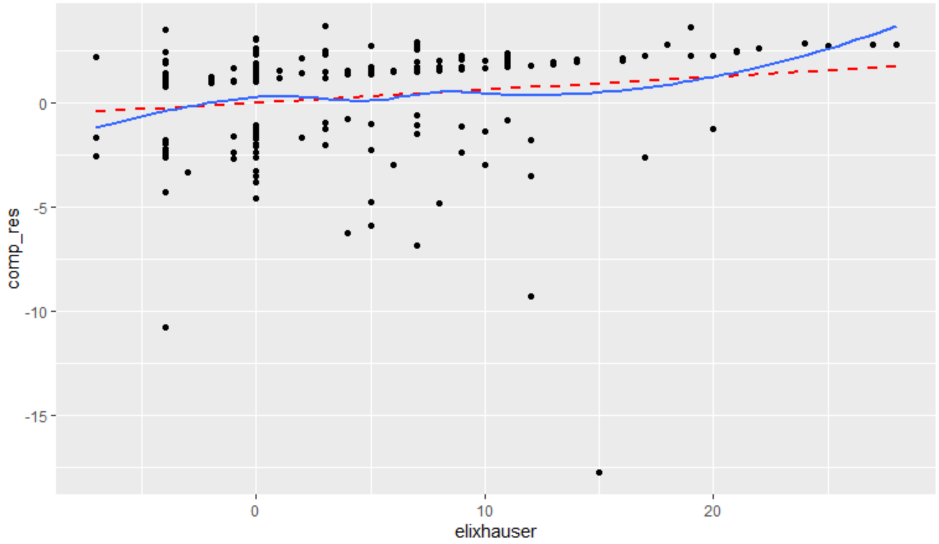
</td></tr>
<tr><td>Age</td><td>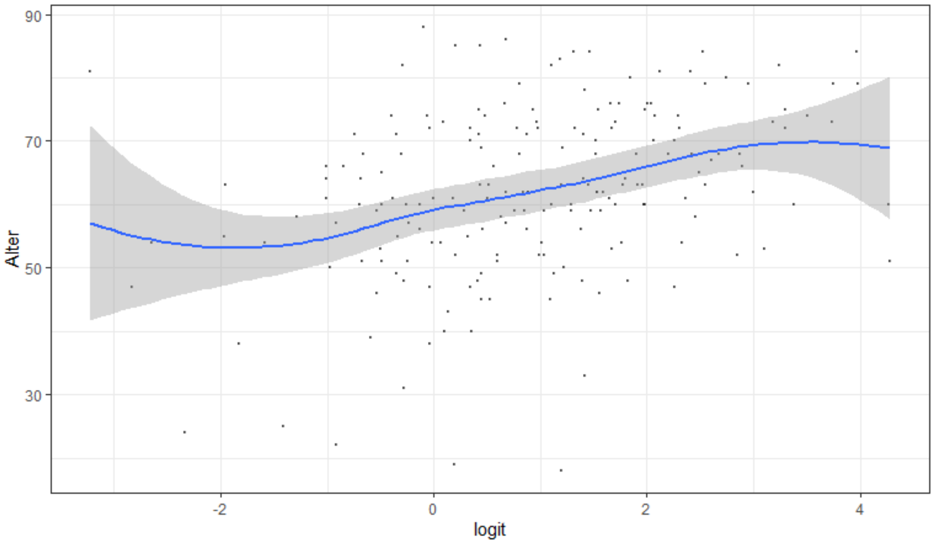

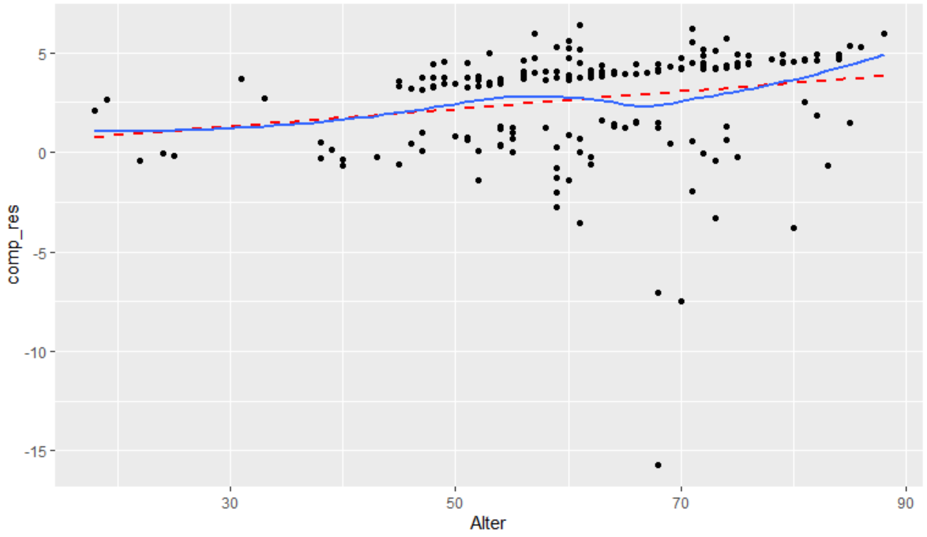
</td></tr>
</table>

Supplementary Table 1 Multiple logistic regression analysis and bootstrapping results for the rehabilitation outcome

	**Global model**			**Selected model**						
	**Beta Coefficient**	**95% CI**	**p-value**	**Beta Coefficient**	**95% CI**	**p-value**	**Bootstrap inclusion frequency (%)**	**Relative conditional bias (%)**	**Bootstrap median**	**Bootstrap 95% CI**
(Intercept)	3.56	-1.05; 8.39	0.137	5.16	2.2; 8.35	<.001	100	41.34	4.97	-0.81; 11.39
**MRC Sum Score at V1**	-0.16	**-0.23; -0.10**	**<.001**	-0.16	**-0.23; -0.10**	**<.001**	100	15.19	-0.18	-0.28; -0.12
**Duration of mechanical ventilation [days]**	0.02	**0.00; 0.03**	**0.019**	0.02	**0.00; 0.03**	**0.011**	88.4	27.3	0.02	0; 0.04
**Acquired brain injury**										
**No**	*Reference*									
**Yes**	**1.55**	**0.22; 3.01**	**0.028**	1.17	0.00; 2.45	0.059	73.5	21.64	1.44	0; 3.43
Sex										
Women	*Reference*									
Men	-0.76	-1.59; 0.02	0.062	-0.80	**-1.57; -0.07**	**0.035**	68.9	37.56	-0.81	-1.77; 0
Age [years]	0.03	0.00; 0.08	0.082	0.02	-0.01; 0.05	0.158	61.2	45.69	0.03	0; 0.08
Diabetes										
No diabetes	*Reference*									
Diabetes	0.69	-0.28; 1.72	0.174	0.80	-0.09; 1.77	0.088	58.8	90.65	0.89	0; 2.27
Elixhauser comorbidity index	0.05	-0.01; 0.11	0.119	0.04	-0.01; 0.09	0.110	56.8	63.46	0.05	0; 0.13
Sepsis										
No	*Reference*									
Yes	0.50	-0.26; 1.28	0.197				41.4	68.84	0	0; 1.36
Preclinical mental health impairment										
None	*Reference*									
Yes	0.52	-0.47; 1.57	0.314				39.8	130.55	0	0; 1.98
Preclinical Frailty										
None-frail	*Reference*									
Frail	0.63	-0.71; 2.15	0.378				37	272.68	0	-0.83; 3.57
Body weight status										
No obesity	*Reference*									
Obesity	0.35	-0.53; 1.25	0.439				34.2	148.52	0	-0.71; 1.60
MoCA at V1	0.02	-0.10; 0.12	0.775				28.8	56.69	0	-0.14; 0.16

Supplementary Table 1 Multiple logistic regression analysis and bootstrapping results for the rehabilitation outcome

	**Global model**			**Selected model**						
	**Beta Coefficient**	**95% CI**	**p-value**	**Beta Coefficient**	**95% CI**	**p-value**	**Bootstrap inclusion frequency (%)**	**Relative conditional bias (%)**	**Bootstrap median**	**Bootstrap 95% CI**
Delirium										
No	*Reference*									
Yes	-0.18	-0.93; 0.58	0.645				23.5	148.67	0	-1.09; 0.76
Living alone										
No	*Reference*									
Yes	-0.15	-1.05; 0.75	0.751				22.8	226.48	0	-1.31; 0.92
ECMO										
No	*Reference*									
Yes	0.22	-0.73; 1.20	0.649				21.5	163.41	0	-0.99; 1.44
Primary diagnose:										
COVID-19	*Reference*									
No	0.07	-0.79; 0.94	0.871				18.1	-125.11	0	-1.06; 0.98
Yes										
CIP/CIM										
No	*Reference*									
Yes	0.01	-0.94; 0.94	0.991				16.9	-542.26	0	-1.12; 1.05
AIC		232.62			217.17					

AIC = Akaike information criterion; 95% CI= 95% Confidence Interval; MRC= Medical Research Council; MoCA=Montreal Cognitive Assessment; V1= Visit 1 at admission to rehabilitation; ECMO= Extracorporeal membrane oxygenation; CIP/CIM=Critical Illness Polyneuropathy / Myopathy. The bootstrap median is zero in case a variable was chosen in <50% of the resamples. Significant values are in bold.

Supplementary Table 2 Model selection frequencies for the model rehabilitation outcome

Model	Included predictors	Count	Percent	Cumulative Percent
1	mrcv1 duration.ventilation brain men age diabetes sepsis Frailty.pre.binary	6	0.6	0.6
2	mrcv1 duration.ventilation brain men age diabetes	5	0.5	1.1
3	mrcv1 duration.ventilation brain men age diabetes elixhauser	5	0.5	1.6
4	mrcv1 duration.ventilation brain men age sepsis Frailty.pre.binary	5	0.5	2.1
5	mrcv1 duration.ventilation men elixhauser	4	0.4	2.5
6	mrcv1 duration.ventilation brain men diabetes elixhauser	4	0.4	2.9
7	mrcv1 duration.ventilation brain men diabetes elixhauser sepsis	4	0.4	3.3
8	mrcv1 duration.ventilation brain men age diabetes elixhauser sepsis	4	0.4	3.7
9	mrcv1 duration.ventilation men age diabetes pre.mental.disease	4	0.4	4.1
10	mrcv1 duration.ventilation brain men diabetes elixhauser sepsis Frailty.pre.binary	4	0.4	4.5
11	mrcv1 duration.ventilation brain men age elixhauser Adipositas moca_v1	4	0.4	4.9
12	mrcv1 duration.ventilation brain men age diabetes elixhauser sepsis	4	0.4	5.3
13	mrcv1 duration.ventilation men diabetes	3	0.3	5.6
14	mrcv1 duration.ventilation brain age diabetes	3	0.3	5.9
15	mrcv1 duration.ventilation brain men age diabetes sepsis	3	0.3	6.2
16	mrcv1 duration.ventilation brain men age elixhauser sepsis	3	0.3	6.5
17	mrcv1 duration.ventilation brain men age elixhauser pre.mental.disease	3	0.3	6.8
18	mrcv1 duration.ventilation brain men diabetes elixhauser pre.mental.disease	3	0.3	7.1
19	mrcv1 duration.ventilation brain men age diabetes elixhauser pre.mental.disease	3	0.3	7.4
20	mrcv1 duration.ventilation brain men age diabetes Frailty.pre.binary	3	0.3	7.7

The model marked in grey is the selected model.

Interpretation of the model stability investigations for the logistic regression for poor rehabilitation outcome:

The model suggested by the bootstrap medians is the same as the selected model, which supports the stability of the model. Bootstrap inclusion frequencies were rather low for age, diabetes and Elixhauser comorbidity scale (57-61%), which is in accordance with rather high relative conditional bias for diabetes (91%) and the Elixhauser scale (63%). Model selection frequencies further added uncertainty for the variables age and the Elixhauser scale, as they were only selected in six of the ten most frequent models. The global shrinkage factor for the selected model was rather low at 0.801 (see Supplementary Table 6 for parameterwise shrinkage factors).

Table 3 Multiple logistic regression analysis for ICUAW at discharge

	Global model			Selected model						
	**Beta Coefficient**	**95% CI**	**p-value**	**Beta Coefficient**	**95% CI**	**p-value**	**Bootstrap inclusion frequency (%)**	**Relative conditional bias (%)**	**Bootstrap median**	**Bootstrap 95% CI**
(Intercept)	0.97	-1.46; 3.42	0.430	1.08	-0.95; 3.16	0.298	100	25.37	1.2	-1.61; 4.38
**Handgrip strength at V1**	-0.07	**-0.10; -0.04**	**<.001**	**-0.07**	**-0.10; -0.05**	**<.001**	100	6.91	-0.08	-0.11; -0.05
**Age [years]**	0.04	**0.01; 0.08**	**0.006**	**0.04**	**0.02; 0.07**	**.004**	92.1	13.05	0.05	0; 0.08
Body weight status										
No obesity	*Reference*									
Obesity	0.90	0.00; 1.85	0.057	0.87	0.01; 1.77	0.052	73.4	33.95	0.98	0; 1.99
Sex										
Women	*Reference*									
Men	-0.80	-1.65; -0.01	0.090	**-0.87**	**-1.68; -0.12**	**0.028**	73	33.63	-0.87	-1.81; 0
**ECMO**										
**No**	*Reference*									
**Yes**	**-1.04**	**-2.05; -0.06**	**0.039**	-0.86	-1.79; 0.06	0.066	73	31.61	-1.08	-2.42; 0
Elixhauser comorbidity index	0.06	-0.01; 0.12	0.089	**0.06**	**0.01; 0.12**	**0.036**	66.2	44.13	0.06	0; 0.14
Acquired brain injury										
No	*Reference*									
Yes	1.08	-0.24; 2.50	0.120	1.06	-0.11; 2.34	0.086	62.2	45.9	1.16	0; 2.56
Preclinical Frailty										
None-frail	*Reference*									
Frail	0.81	-0.75; 2.66	0.341				40	184.4	0	-0.02; 3.91
CIP/CIM										
No	*Reference*									
Yes	0.43	-0.46; 1.33	0.339				32	121.13	0	0; 1.40
Delirium										
No	*Reference*									
Yes	-0.25	-1.02; 0.52	0.525				26	163.42	0	-1.21; 0.65
Sepsis										
No	*Reference*									
Yes	-0.21	-1.02; 0.59	0.616				23.8	232.76	0	-1.34; 0.67

Table 3 Multiple logistic regression analysis for ICUAW at discharge

	Global model			Selected model						
	**Beta Coefficient**	**95% CI**	**p-value**	**Beta Coefficient**	**95% CI**	**p-value**	**Bootstrap inclusion frequency (%)**	**Relative conditional bias (%)**	**Bootstrap median**	**Bootstrap 95% CI**
Duration of mechanical ventilation [days]	0.00	-0.01; 0.01	0.936				23	-358.62	0	-0.02; 0.02
Primary diagnose: COVID-19	*Reference*									
No	0.04	-0.84; 0.93	0.937				22	-523.2	0	-1.14; 1.11
Yes										
Diabetes										
No	*Reference*									
Yes	-0.26	-1.24; 0.74	0.604				20.3	196.6	0	-1.28; 0.68
AIC		218.58			207.59					

AIC = Akaike information criterion; 95% CI= 95% Confidence Interval; MRC= Medical Research Council; MoCA=Montreal Cognitive Assessment; V1= Visit 1 at admission to rehabilitation; ECMO= Extracorporeal membrane oxygenation; CIP/CIM=Critical Illness Polyneuropathy / Myopathy. The bootstrap median is zero in case a variable was chosen in <50% of the resamples. Significant values are in bold.

Supplementary Table 4 Model selection frequencies for model ICUAW at discharge

Model	Included predictors	Count	Percent	Cumulative Percent
1	gripstrength.% age obesity men ecmo elixhauser brain	24	2.4	2.4
2	gripstrength.% age obesity men elixhauser brain	15	1.5	3.9
3	gripstrength.% age obesity men ecmo elixhauser	13	1.3	5.2
4	gripstrength.% age obesity men ecmo elixhauser brain Frailty.pre.binary	12	1.2	6.4
5	gripstrength.% age obesity men ecmo elixhauser brain Frailty.pre.binary delirium	11	1.1	7.5
6	gripstrength.% age obesity ecmo Frailty.pre.binary	9	0.9	8.4
7	gripstrength.% age obesity men ecmo elixhauser brain sepsis	9	0.9	9.3
8	gripstrength.% age obesity men elixhauser brain cipcim	8	0.8	10.1
9	gripstrength.% age obesity men ecmo elixhauser sepsis	8	0.8	10.9
10	gripstrength.% age obesity men elixhauser brain duration.ventilation	8	0.8	11.7

Supplementary Table 4 Model selection frequencies for model ICUAW at discharge

Model	Included predictors	Count	Percent	Cumulative Percent
11	gripstrength.% age obesity men ecmo elixhauser brain duration.ventilation	8	0.8	12.5
12	gripstrength.% age men ecmo elixhauser brain	7	0.7	13.2
13	gripstrength.% age obesity men ecmo elixhauser brain delirium	7	0.7	13.9
14	gripstrength.% age obesity men elixhauser brain	7	0.7	14.6
15	gripstrength.% age obesity men ecmo elixhauser brain Frailty.pre.binary cipcim delirium	6	0.6	15.2
16	gripstrength.% age obesity men ecmo elixhauser cipcim sepsis	6	0.6	15.8
17	gripstrength.% age obesity men ecmo elixhauser brain covid	6	0.6	16.4
18	gripstrength.% age obesity men ecmo elixhauser brain	6	0.6	17
19	gripstrength.% age obesity men ecmo brain	5	0.5	17.5
20	gripstrength.% age obesity men elixhauser brain Frailty.pre.binary	5	0.5	18

The model marked in grey is the selected model.

Supplementary Table 5 Parameterwise shrinkage factors

**Model poor rehabilitation outcome**		**Model ICUAW**	
**Variable**	**Parameterwise shrinkage factor**	**Variable**	**Parameterwise shrinkage factor**
MRC sum score	0.916	Handgrip strength	0.846
Duration of mechanical ventilation	0.712	Age	0.753
Sex	0.569	Sex	0.646
Brain injury	0.492	Elixhauser comorbidity scale	0.655
Diabetes	0.572	Obesity	0.583
Elixhauser comorbidity scale	0.513	ECMO	0.689
Age	0.433	Brain injury	0.541

Interpretation of the model stability investigations for the logistic regression for ICUAW at discharge:

Bootstrap inclusion frequencies were ≥ 60% for all variables of the selected model, thus the model suggested by the bootstrap medians was the same as the selected model, which supports the final model. Only the slightly increased relative conditional biases of the Elixhauser scale and brain injury (44-46%) indicate some uncertainty. Model selection frequencies (Supplementary Table 5) supported the stability of the final model, as it was the most frequently chosen model by the bootstrapping procedure. The global shrinkage factor for the selected model was rather low at 0.797 (Supplementary Table 6).
